# Simultaneous Determination of Moxifloxacin and Flavoxate by RP-HPLC and Ecofriendly Derivative Spectrophotometry Methods in Formulations

**DOI:** 10.3390/ijerph16071196

**Published:** 2019-04-03

**Authors:** Mahesh Attimarad, Muhammad Shahzad Chohan, Abdulmalek Ahmed Balgoname

**Affiliations:** 1 Department of Pharmaceutical Sciences, College of Clinical Pharmacy, King Faisal University, Al-Ahsa 31982, Saudi Arabia; a.balgoname@gmail.com; 2 Department of Biomedical Sciences, College of Clinical Pharmacy, King Faisal University, Al-Ahsa 31982, Saudi Arabia; chohanshahzad@hotmail.com

**Keywords:** determination, ecofriendly, flavoxate, HPLC, moxifloxacin, ratio first derivative, spectrophotometry, validation

## Abstract

Simple, fast, and precise reversed-phase (RP)-high-performance liquid chromatography (HPLC) and two ecofriendly spectrophotometric methods were established and validated for the simultaneous determination of moxifloxacin HCl (MOX) and flavoxate HCl (FLX) in formulations. Chromatographic methods involve the separation of two analytes using an Agilent Zorbax SB C18 HPLC column (150 mm × 4.6 mm; 5 µm) and a mobile phase consisting of phosphate buffer (50 mM; pH 5): methanol: acetonitrile in a proportion of 50:20:30 v/v, respectively. Valsartan was used as an internal standard. Analytes were monitored by measuring the absorbance of elute at 299 nm for MOX and 250 nm for FLX and valsartan. Two environmentally friendly spectrophotometric (first derivative and ratio first derivative) methods were also developed using water as a solvent. For the derivative spectrophotometric determination of MOX and FLX, a zero-crossing technique was adopted. The wavelengths selected for MOX and FLX were −304.0 nm and −331.8 nm for the first derivative spectrophotometric method and 358.4 nm and −334.1 nm for the ratio first-derivative spectrophotometric method, respectively. All methods were successfully validated, as per the International Conference on Harmonization(ICH) guidelines, and all parameters were well within acceptable ranges. The proposed analytical methods were successfully utilized for the simultaneous estimation of MOX and FLX in formulations.

## 1. Introduction

**Figure 1:**
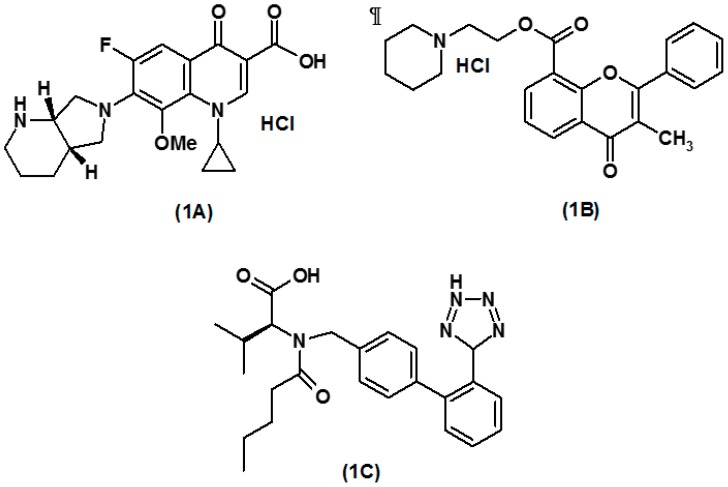
Chemical structures of moxifloxacin HCl [1A], Flavoxate HCl [1B] and valsartan [1C].

Moxifloxacin hydrochloride (MOX; Figure 1A) is a broad-spectrum fluoroquinolone antibacterial agent used in the treatment of eye, respiratory tract, lung, and urinarytract infections, and it is also used to treat skin allergies, pneumonia, and abdominal bacterial infections. It is highly effective against Gram-positive microorganisms and shows moderate activity against Gram-negative microorganisms and anaerobes. [1,2]. Older fluoroquinolones are structurally modified to generate MOX, so it can be effective against many beta lactam-resistant pathogens. MOX acts by strongly inhibiting two enzymes: DNA gyrase and topoisomerase IV, which are essential for the replication of bacteria, as well as for the transformation, restoration, and rearrangement of DNA, leading to bacterial death [3,4,5,6]. Flavoxate hydrochloride (FLX; Figure 1B) is a flavone derivative that acts as a strong smooth-muscle relaxant. Different mechanisms of action are reported for FLX, as it acts as a calcium antagonist, phosphodiesterase enzyme inhibitor, and local anesthetic. FLX shows inhibitory action on the contraction of the human bladder, as produced by muscarinic receptors; thus, it is used in the treatment of overactive bladder syndrome to relieve symptomatic pain, urinary frequency, and other inflammatory disorders of the urinary tract [7,8,9,10].

A literature review revealed that many analytical methods such as direct [11,12,13] and derivative [14] ultraviolet-visible (UV-Vis) spectrophotometric, spectrofluorometric [15,16], high-performance liquid chromatography (HPLC) [17,18,19,20,21], ultra-performance liquid chromatography (UPLC) [22], liquid chromatography (LC)-mass spectrometry (MS) [23,24], and capillary zone electrophoresis [25,26] methods for the determination of MOX alone and in combination with other drugs in different formulations and in different biological samples. Bibliography data showed that UV-Vis spectrophotometric [27], HPLC [28,29,30,31,32,33,34], electroanalytical [35], and capillary electrophoresis methods [36] were reported for the determination of FLX alone or in combination with other drugs. Only one reversed-phase (RP)-HPLC method has been reported for the concurrent estimation of MOX and FLX in formulation [37]. However, this method does not use an internal standard and requires a long analysis time. The UV spectra of MOX and FLX showed complete band overlap, which makes it difficult to simultaneously determine both analytes from co-formulations without prior separation. Hence, RP-HPLC and derivative spectroscopy methods, such as first derivative and first derivatives of the ratio spectra, were utilized to avoid the spectral interference that results from band overlap. 

HPLC is the most extensively used analytical technique for quality controlof drugs due to its high efficiency, selectivity, reproducibility and sensitivity. Further, the current trend is to develop simple, rapid, and ecofriendly analytical methods to save the environment. Hence, in this proposal, an effort was made to establish a simple, fast, accurate, precise, and specific RP-HPLC method, as well as simple, ecofriendly, derivative UV spectrophotometric procedures to simultaneously estimate MOX and FLX from formulations. All required parameters were validated, as per the International Conference on Harmonization (ICH) guidelines for all three methods, and they were effectively used for the simultaneous estimation of MOX and FLX in solid dosage form.

## 2. Experimental Methods

### 2.1. Chemicals

Pure samples of MOX hydrochloride (99.3%), FLX hydrochloride (99.5%), and internal standard valsartan (VST; Figure 1C) were purchased from Biokemix India Ltd. (Telangana, India). Solvents and reagents used for the experiments were either of HPLC or analytical grade. HPLC-grade methanol and acetonitrile were purchased from Sigma Aldrich Co. (St. Louis, MO, USA). Potassium dihydrogen phosphate and dipotassium hydrogen phosphates were purchased from Scharlau S.L. (Barcelona, Spain). Pure distilled water prepared using Millipore was used throughout the experiments (EMD Millipore, Billerica, MA, USA). A tablet containing 400 mg of MOX and 200 mg of FLX was purchased from the local market.

### 2.2. Instruments

Chromatography was performed on an HPLC system (Shimadzu Prominence Liquid Chromatography, Tokyo, Japan) equipped with an isocratic pump (LC-20AT), auto-sample injector (SIL20A), UV-Vis detector (SPD-20A), and column oven (CTO-20A). Analyte peaks were monitored using the Shimadzu software, LC solutions. The pH level of the mobile phase was adjusted using an Omega PHH 222 (Stamford, CT, USA) pH meter.

Spectrophotometric methods were developed using a Shimadzu UV-Vis spectrophotometer (1600) with 10 mm Quartz cuvettes. The slit width was adjusted to 1 nm and the samples were scanned at a speed of 50 nm/min. The ratio and derivative spectra were computed using Shimadzu UV probe software (version 2.21) (Shimadzu, Tokyo, Japan).

### 2.3. Preparation of Standard Stock and Working Solutions

#### 2.3.1. HPLC Method

Standard stock solutions of MOX, FLX, and VST were arranged by individually dissolving precisely weighed (100 mg) MOX, FLX, and VST into 50 mL of methanol using a 100 mL volumetric flask. Further, a final volume of 100 mL was reached using methanol. Standard stock solutions were kept in a refrigerator at 4 °C. Further, working standards were prepared daily by diluting these solutions to a required concentration with the mobile phase. 

#### 2.3.2. Derivative Spectrophotometric Method

Standard stock solutions of MOX and FLX were arranged by separately dissolving exactly weighed (20 mg) MOX and FLX into 50 mL of water using a 100 mL volumetric flask. Volumetric flasks were sonicated for 10 min to completely dissolve the analytes; the volume was finalized up to mark using water.

### 2.4. Preparation of MOX and FLX Sample Solutions using Tablets

Twenty tablets comprising 400 mg of MOX and 200 mg of FLX were weighed and the average weight was calculated; the tablets were subsequently powdered. For HPLC, the tablet powder corresponding to 100 mg of MOX and 50 mg of FLX was weighed and dissolved in 50 mL of methanol present in a 100 mL volumetric flask. For spectrophotometric methods, tablet powder equivalent to 20 mg of MOX and 10 mg of FLX was weighed and dissolved in 50 mL of water present in a 100 mL volumetric flask. Volumetric flasks were sonicated for 20 min to fully extract analytes into the solvent and they were then filtered. The volume was increased up to the mark using methanol and water, respectively. For HPLC, the required amount of mobile phase was added to the above solutions to achieve a concentration in the range of the calibration curve, and an internal standard concentration was maintained at 50 µg/mL. For spectrophotometric methods, solutions were diluted with water to achieve a concentration in the range of the calibration curve.

## 3. Procedures

### 3.1. HPLC Method: Chromatographic Conditions

Analytes were chromatographically separated on a reversed-phase Agilent Zorbax SB C_18_ HPLC column (150 mm × 4.6 µm internal diameter with a 5 µm particle size) using methanol, acetonitrile, and 50 mM of phosphate buffer (the pH level was adjusted to 5 with orthophosphoric acid) at a ratio of 20:30:50 v/v as the mobile phase. The mobile phase was prepared daily, filtered using a Millipore membrane filter (0.45 µm), and degassed before use. An isocratic method was developed with a flow rate of 1.2 mL/min and the column temperature was kept at 25 °C. The detector wavelength was set to 299 nm for MOX and changed online after 2.5 min to 250 nm for FLX and the internal standard. Then, 20 µL of the analyte solution was injected into the HPLC for analysis.

### 3.2. Calibration Curve Construction

An essential quantity of stock solutions of MOX and FLX, as well as internal standard solutions were transferred into 10 mL volumetric flasks. Then, the solutions were diluted with the mobile phase to achieve a concentration of MOX and FLX in the range of 5–200 µg/mL and 2–200 µg/mL, respectively, and to achieve a concentration of 50 µg/mL for the internal standard. In all, 20 µL of each solution was injected and the analytes were eluted using optimized HPLC analysis conditions in triplicate. The peak area of the analytes was recorded and a ratio of peak area of MOX and FLX to a peak area of the internal standard was computed separately. The average peak area ratio of MOX and FLX was plotted against the concentration of analytes to obtain calibration curves. Further, corresponding regression equations were constructed from the calibration curves.

### 3.3. Application of the HPLC Method to the Formulation

The formulation sample was diluted with mobile phase to achieve concentrations of 40 µg/mL, 80 µg/mL, and 120 µg/mL of MOX and 20 µg/mL, 40 µg/mL, and 60 µg/mL of FLX. Then, 20 µL of solution was injected after adding the internal standard solutions and eluted with the optimized mobile phase under optimized HPLC conditions. From the ratio of the peak area, the tablet contents of MOX and FLX were calculated using regression equations. The pre-analyzed sample solutions were also used for recovery studies.

### 3.4. Spectrophotometric Method: Construction of the Calibration Curve by First-Derivative Spectrophotometry

The working standard solutions were prepared by transferring a sufficient amount of standard stock solutions of MOX and FLX into separate 10 mL volumetric flasks. A final volume was reached using water to obtain six different concentrations corresponding to 2, 4, 8, 12, 16 and 20 µg/mL of FLX and six different concentrations corresponding to 1, 2, 4, 8, 12 and 16 µg/mL of MOX separately. The UV spectra were recorded in the range of 200–400 nm using water as a blank solution. With the help of UV probe software the absorption spectra were converted into first-derivative spectra using 4 nm as *dλ*, to get a set of six spectra (*dA/dλ* vs wavelength) for MOX and set of six spectra (*dA/dλ* vs wavelength) for FLX. The amplitude of *dA/dλ* peak was measured at −304.0 nm for MOX (zero crossing of FLX) and at −331.8 nm for FLX (zero crossing of MOX); the linearity graphs were plotted against concentration, and the corresponding regression equations were constructed from the calibration curves.

### 3.5. Construction of the Calibration Curve by Ratio First-Derivative Spectrophotometry

Next, 4 µg/mL solutions of MOX and FLX were prepared separately and UV absorption spectra were recorded in the range of 200–400 nm. Similarly, six different concentrations, corresponding to 1, 2, 4, 8, 12 and 16 µg/mL each of MOX and FLX were prepared and UV absorption spectra were recorded. Then, with the help of UV probe software, the ratio spectra were computed by dividing each spectrum of mixture of MOX and FLX with the absorption of spectra for 4 µg/mL of FLX to get a set of six ratio spectra for MOX. Similarly, the FLX ratio spectra were computed by dividing each spectrum of mixture of MOX and FLX with the absorption of spectra for 4 µg/mL of MOX to get a set of six ratio spectra for FLX. Then, the first-derivative spectra were figured from ratio spectra using 4 nm as *dλ* to get a set of six first derivative spectra for MOX (*dA/dλ* vs. wavelength) and set of 5 spectra for FLX (*dA/dλ* vs. wavelength) separately. The amplitude of *dA/dλ* peak was measured at 358.4 nm for MOX and at −334.1 nm for FLX; graphs were plotted against concentration, and the corresponding regression equations were constructed from the calibration curves.

### 3.6. Application of First Derivative and Ratio First-derivative Spectrophotometry to the Formulations

An aliquot of tablet solution was diluted with water in a volumetric flask to obtain a concentration in the range of the calibration curve. For first-derivative spectra, the solutions were scanned to record UV absorption spectrum in the range of 200–400 nm and converted into first-derivative spectrum using 4 nm as *dλ*. The amplitude of *dA/dλ* peak was measured at −304.0 nm for MOX (zero crossing of FLX) and at −331.8 nm for FLX (zero crossing of MOX); and concentration of MOX and FLX were calculated using corresponding regression equations. For the ratio first-derivative spectra, the UV absorption spectra were divided by the absorption spectra of 4 µg/mL of MOX and FLX separately and resulting ratio spectra were converted into the first-derivative spectra using four nm as *dλ* to get first derivative spectra of MOX and FLX separately. The concentrations of MOX and FLX were calculated by determining the amplitude of *dA/dλ* peak at 358.4 nm for MOX and at −334.1 nm for FLX; as described in the aforementioned procedure, and from corresponding regression equations.

## 4. Results and Discussion

### 4.1. HPLC Method Development and Optimization

All important HPLC conditions that induced the chromatographic separation of MOX and FLX, along with the internal standard, were optimized to obtain high-resolution spectra with good peak shape and accurate quantitative estimation of MOX and FLX. 

**Figure 2:**
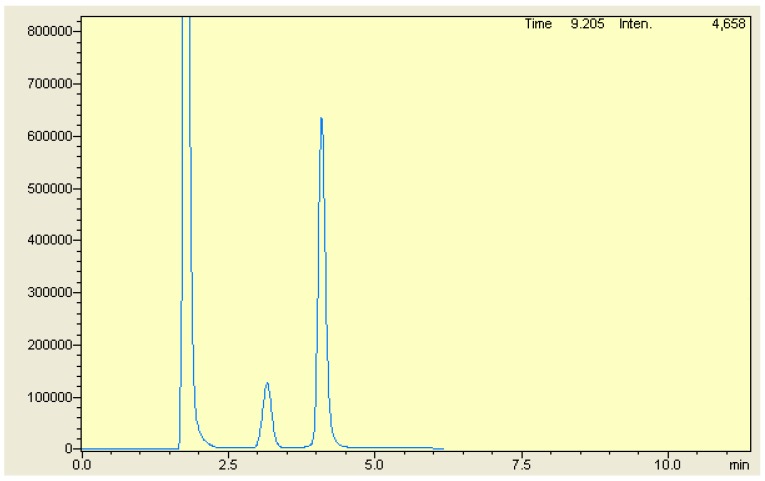
Typical Chromatogram of MOX [1.77 min], VST [3.22 min] and FLX [4.26 min].

To obtain good chromatographic separation and high-resolution spectra for MOX and FLX, along with the internal standard, different RP-HPLC columns and different proportions of the most common HPLC solvents, methanol, acetonitrile, water, or buffer as a mobile phase were tried. Good separation of both drugs along with the internal standard was achieved by isocratic elution using an Agilent Zorbax SB C_18_ HPLC column (150 mm × 4.6 mm; 5 µm internal diameter). The mobile phase composition, along with pH level and the buffer concentration, were optimized (Supplementary material 1). Several combinations of the mobile phase were tried to rapidly obtain complete separation. The mobile phase consisting of methanol: acetonitrile: phosphate buffer (50 mM; pH adjusted to 5 with orthophosphoric acid) in a ratio of 20:30:50 v/v was found to be optimal for complete baseline separation for all analytes featuring a high resolution and good peak shape with an appropriate tailing factor. MOX demonstrated maximum absorption at a wavelength of 299 nm, whereas FLX and the internal standard showed λmax at 250 nm in the mobile phase as a solvent; hence, the detector was set at 299 nm to estimate MOX. After 2.5 min, the wavelength was changed online to 250 nm for FLX and the internal standard. Flow rates of 0.5 mL/min, 0.8 mL/min, 1.0 mL/min, 1.2 mL/min, and 1.5 mL/min were tried. With a flow rate of 1 mL/min, less peak broadening was observed over a long analysis time. With 1.5 mL/min, MOX was eluted at dead volume and the column back pressure was high. A flow rate of 1.2 mL/min was found to be ideal for good base line separation with a short analysis time. Figure 2 shows the typical chromatogram of analytes. All three analytes were completely separated with a high resolution within 5 min. 

### 4.2. Spectrophotometric Method

**Figure 3:**
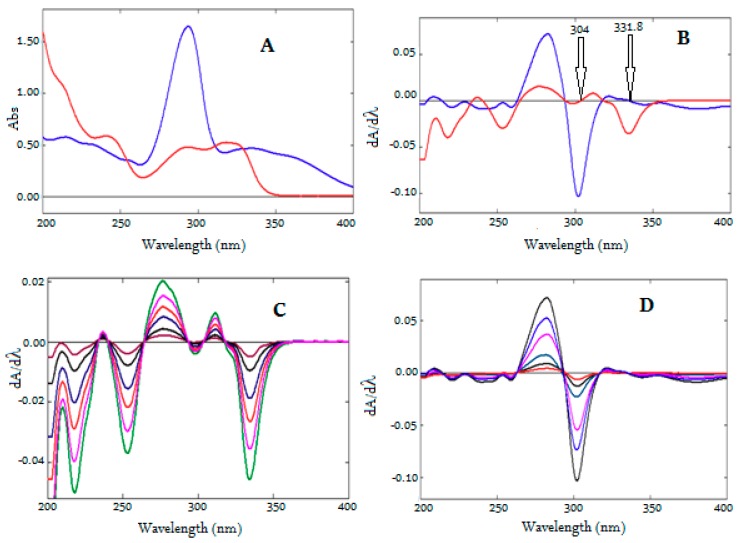
UV spectra of MOX and FLX (**A**), Overlain First derivative UV spectra of MOX and FLX (**B**), First derivative UV spectra of FLX (**C**, 2–20 µg/mL), First derivative UV spectra of MOX (**D**, 1–16 µg/mL).

Two derivative spectrophotometric methods were established for the simultaneous determination of MOX and FLX in formulation. Both analytes were soluble in water and showed good UV absorption. Hence, water was selected as a solvent to develop a simple, accurate, and ecofriendly analytical method. However, UV spectra of both analytes showed complete overlap (Figure 3A), making it difficult to estimate either of the analytes in the presence of each other without prior separation. Hence, the derivative spectroscopy technique was adopted, as it permits the simultaneous estimation of multicomponent formulations when absorbance was measured at the zero-crossing point. At the zero-crossing point, one of the analytes had zero amplitude and the other showed absorbance, even at different concentrations [38,39]. Another simple ratio derivative spectroscopic method reported by Salinas et al. [40] for binary mixture was adopted for simultaneous determination of MOX and FLX. In this method, the absorption spectra of mixture of analytes were divided by the absorption spectrum of one analyte and the resulted ratio spectrum is converted into first derivative spectrum. First derivative of the ratio spectrum allow us to measure analytical signals at different wavelengths with several maxima and minima and determine the concentration of active components in presence of probably interfering another drug and excipients of formulation [41].

For the first-derivative spectroscopic method, the solutions of MOX and FLX were scanned to record UV absorption spectra using water as blank and the spectra were converted into first-derivative spectra with the help of Shimadzu UV probe software.Wavelengths of 2 nm, 4 nm, 6 nm, 8 nm, and 10 nm were tried as *dλ;* 4nm *dλ* was found to be optimal, and 4 nm *dλ* was thus selected to generate first-derivative spectra (*dA/dλ* vs. wavelength). The overlain first derivative spectra (Figure 3B) of MOX and FLX showed, two minima wavelengths at −214.2 nm and −304.0 nm, where MOX demonstrated some absorption and FLX showed zero crossing. However, the peak height of *dA/dλ* spectra at −214.2 was lower compared to the amplitude at −304.0 nm. Whereas the first-derivative spectra of FLX showed two minima wavelengths at −215.4 nm and −331.8 nm, where MOX showed zero crossing. However, the amplitude of *dA/dλ* spectra at −215.4 nm was lower when compared to the amplitude at −331.8 nm. Hence, wavelengths −304.0 nm and −331.8 nm were selected and first derivative spectra were constructed forMOX and FLX. Figure 3C,D showed the first derivative spectra of FlXandMOX, respectively. The amplitudes of *dA/dλ* spectra were measured at −304.0 nm and −331.8 nm (Figure 3C,D) at different concentrations of MOX and FLX, respectively, and calibration curves were constructed. Alternatively, regression equations were computed from the calibration curves (Supplementary material 2).

**Figure 4:**
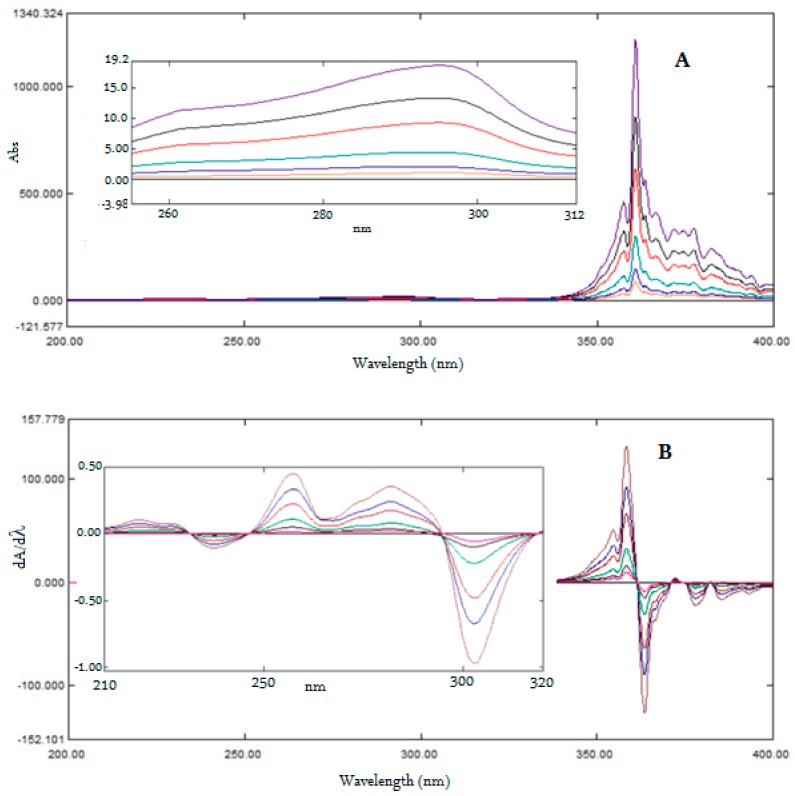
Ratio UV spectra MOX (1–16 µg/mL) using 4 µg/mL solution spectra of FLX as devisor (**A**), First derivative of ratio spectra of MOX (1–16 µg/mL, **B**) with enlarged part for 200 to 320 nm.

**Figure 5:**
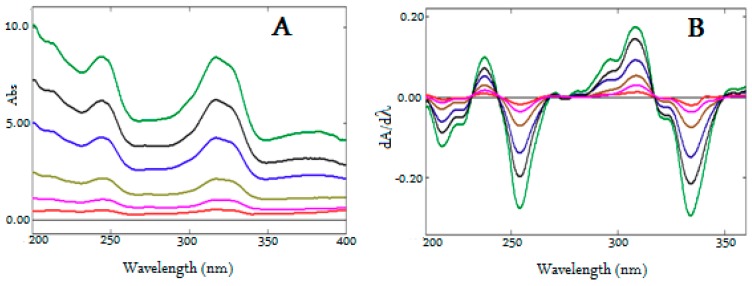
Ratio UV spectra FLX (1–16 µg/mL) using 4 µg/mL solution spectra of MOX as devisor (**A**), First derivative of ratio spectra of FLX (1–16 µg/mL, **B**).

For ratio first-derivative spectroscopy, UV absorption spectra were obtained for a mixture of MOX and FLX in the concentration range of 1−16 µg/mL and were divided by the absorption spectra of standard solutions of MOX and FLX, separately to get set of six ratio spectra for MOX (Figure 4A) and six spectra for FLX (Figure 5A). Different concentrations in the range of 1–20 µg/mL were tried; 4 µg/mL was found to be optimal because the peak amplitude showed good linearity and good recovery from the mixed sample solutions in the laboratory. The resulting ratio spectra were converted into first-derivative spectra using 4 nm as *dλ* to get first derivative of ratio spectra for MOX (Figure 4B) and first derivative of ratio spectra for FLX (Figure 5B).

The ratio first-derivative spectra of MOX (Figure 4B) showed three maxima at 256.9 nm, 281.8 nm, and 358.4 nm, and four minima at −368.6 nm, −378.2 nm, −385.7 nm, and −302.7 nm. The ratio first-derivative spectra of FLX (Figure 5B) showed two maxima at 237.7 nm and 307.7 nm, and three minima at −218.0 nm, −253.6 nm, and −334.1 nm. Further, the amplitudes of *dA/dλ* peaks measured at these wavelengths were proportional to the concentration. For the simultaneous estimation of MOX and FLX from the standard solutions and formulations, wavelengths 358.4 nm and −334.1 nm were selected, respectively, because the mean recovery of MOX and FLX in the mixed samples in the laboratory was found to be suitable with a low standard deviation. Figure 4B and Figure 5B showed the ratio first-derivative spectra of MOX and FLX at six different concentrations corresponding to 1, 2, 4, 8, 12 and 16 µg/mL. The amplitudes of *dA/dλ* peaks were measured at 358.4 nm and −334.1 nm for MOX and FLX, respectively, and calibration curves were constructed. Alternatively, regression equations were computed from the calibration curves. (Supplementary Materials).

### 4.3. Method Validation

The proposed HPLC and derivative spectroscopic methods were validated as per the ICH guidelines.

### 4.4. Linearity

**Figure 6:**
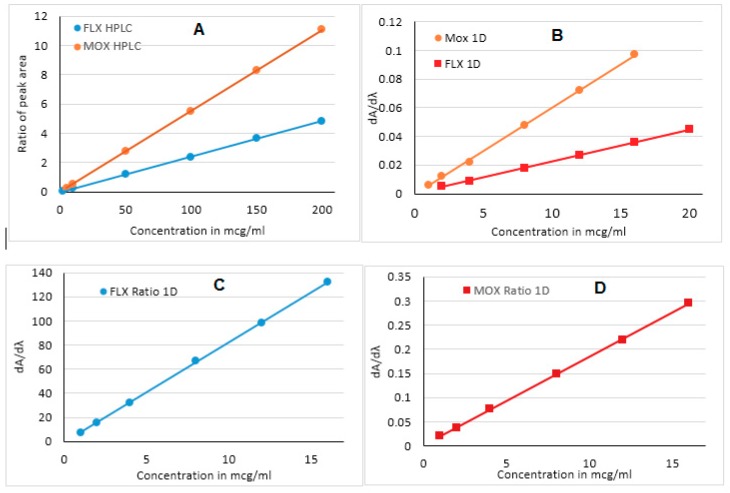
Standard Calibration curve for MOX and FLX by RP HPLC(**A**), First derivative (**B**), and ratio first derivative UV spectrophotometry (**C**,**D**).

A calibration curve was drawn in the range of 5–200 µg/mL for MOX and 2–200 µg/mL for FLX in the proposed RP-HPLC method (Figure 6A). Calibration curves showed perfect linearity in this concentration range with a good regression coefficient (*r^2^* > 0.998). In the first-derivative UV spectroscopy, linearity was constructed in the range of 1–16 µg/mL for MOX and 2–20 µg/mL for FLX, (Figure 6B) whereas both analytes showed good linearity in the range of 1–16 µg/mL for both MOX and FLX in the ratio first-derivative spectroscopy with a good correlation coefficient (Figure 6C and 6D). The linearity range, regression equations, and coefficients aretabulated in Table 1. The limits of detection and quantification were also calculated in all three methods using the 3.3 σ/s and 10 σ/s criteria, respectively, where σ signifies the standard deviation of the response and s signifies the slope of the calibration curve (Table 1).

### 4.5. Precision and Accuracy

For the HPLC method, repeatability was determined by injecting 20 µL of freshly prepared 5 µg/mL, 100 µg/mL, and 200 µg/mL solutions of MOX and 2 µg/mL, 100 µg/mL, and 200 µg/mL solutions of FLX along with 50 µg/mL of the internal standard. For first-derivative UV spectroscopy, repeatability was determined using 1 µg/mL, 8 µg/mL, and 16 µg/mL for MOX and 2 µg/mL, 10 µg/mL, and 20 µg/mL for FLX, whereas the ratio first-derivative was determined at 1 µg/mL, 8 µg/mL, and 16 µg/mL for MOX and FLX. Analysis of all solutions was carried out in triplicate on the same day using optimized experimental conditions. The percent relative standard deviations and percent relative errors were calculated and found to be within acceptable ranges (Table 2).

Intermediate precision was also determined for the solutions covering the entire calibration range and using all three methods on 3 successive days. The percent relative standard deviation and percent relative errors were calculated and found to be within standard ranges, representing the good precision and accuracy of the proposed methods.

### 4.6. Recovery Studies

A recovery study was executed using the standard addition method. For the HPLC method, 40 µg/mL, 80 µg/mL, and 120 µg/mL of MOX and 20 µg/mL, 40 µg/mL, and 60 µg/mL of FLX, along with 50 µg/mL of the internal standard were added to the previously analyzed formulation solution (80 µg/mL of MOX and 40 µg/mL of FLX) and analyzed using the optimized HPLC method. The mean percent recovery of the added amount was found to be 99.28% for MOX and 98.73% for FLX.

For the first derivative, a UV spectroscopy recovery study was carried out at three concentrations (3 µg/mL, 6 µg/mL, and 9 µg/mL) of MOX and (1.5 µg/mL, 3 µg/mL, and 4.5 µg/mL), of FLX, which were added to the previously analyzed formulation solution (6 µg/mL of MOX and 3 µg/mL of FLX). For the ratio first-derivative method, a recovery study was determined at 4 µg/mL, 6 µg/mL, and 8 µg/mL of MOX and FLX, which were added to the previously analyzed formulation solution (8 µg/mL of MOX and 4 µg/mL of FLX). The mean recovery ranged from 99.43% to 99.72% for MOX and 98.85% to 99.48% for FLX, indicating the excellent recovery of the proposed methods (Table 3).

### 4.7. Specificity

**Figure 7:**
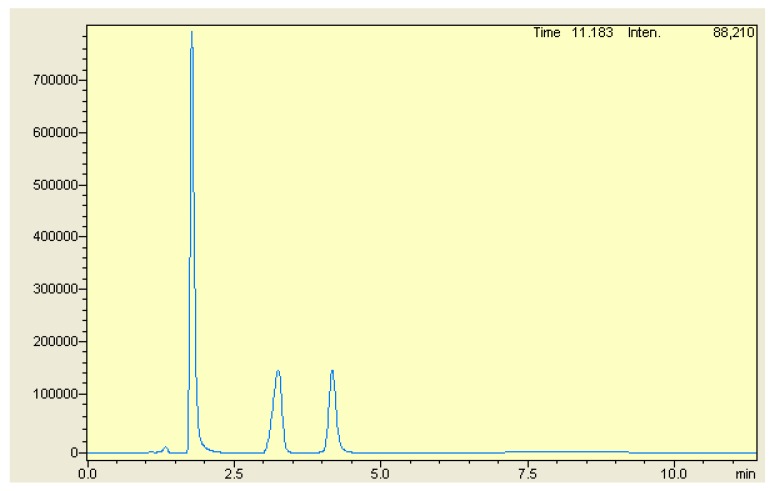
Chromatogram of formulation; MOX (1.76 min), VST (50 µg/mL, 3.24 min), and FLX (4.25 min).

Specificity of the HPLC method was established by comparing the chromatograms of the standard and formulation solutions. No interfering peaks were witnessed at the position of the analyte peaks. (Figure 7) The results of the recovery studies using all three methods also indicated the absence of formulation-excipient interference.

### 4.8. Application to Formulations

**Figure 8:**
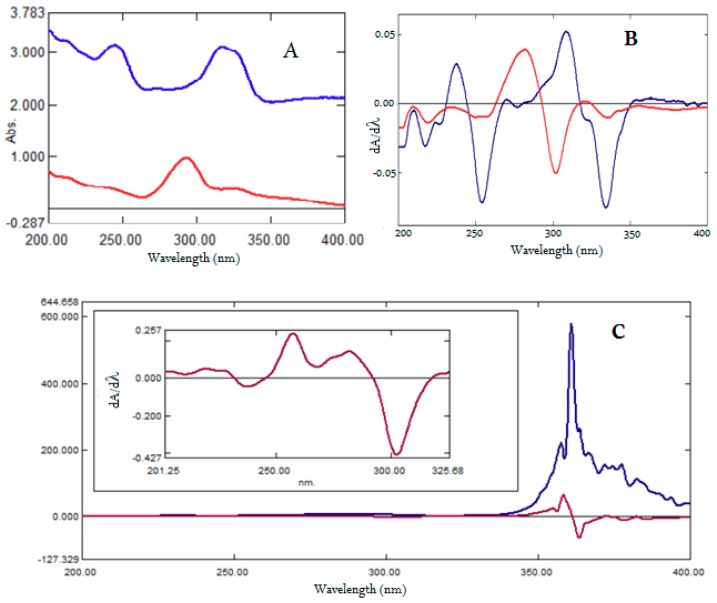
UV absorption spectra of formulation (**A**, Red), first derivative UV spectra of formulation (**B**, Red). Ratio spectra (**A**, Blue) and first derivative of ratio spectra (**B**, Blue) of formulation for FLX using four µg/mL solution spectra of MOX as devisor. Ratio spectra (**C**, Blue) and first derivative of ratio spectra (**C**, Brown) of formulation for MOX using four µg/mL solution spectra of FLX as devisor, with enlarged part of ratio first derivative spectrum from 201 nm to 325 nm.

The proposed methods were successfully utilized for the simultaneous quantification of MOX and FLX from the tablet formulations. (Figure 7 and Figure 8) The assay results of HPLC, firstand ratio firstderivative methods were found to be 98.67%, 99.18% and 101.29% for MOX, and 99.03%, 100.89% and 98.47% for FLX respectively. The findings from the analysis report were in good agreement with the label claim. The validity of the developed methods was evaluated via recovery studies using the standard addition method. Assay results with a low percentage relative standard deviation indicated the good accuracy and excellent precision of the proposed methods for the analysis of MOX and FLX from the formulations (Table 3).

## 5. Conclusions

The proposed methods are simple, fast, and accurate for the simultaneous estimation of MOX and FLX from the formulations; hence, these methods can be successfully used for the regular analysis of MOX and FLX for quality control. The advantages of the HPLC method include the fact that analysis can be performed within five min, and it features good baseline separation, symmetric peaks, and a wider concentration range. UV spectrophotometric methods were simple, economical, and ecofriendly, as water was used as the solvent. 

## Figures and Tables

**Table 1:** Regression equations and validation parameters results for MOX and FLX.

Parameters	HPLC		First Derivative UV		Ratio First Derivative UV	
Drugs	MOX	FLX	MOX	FLX	MOX	FLX
Retention Time ± %RSD	1.77 ± 0.03	4.26 ± 0.04				
Wave length [nm]	299	250	304.0	−331.8	358.4	−334.1
Linearity Range [µg/mL]	5–200	2–200	1–16	2–20	1–16	1–16
LOD [µg/mL]	0.45	0.6	0.22	0.35	0.26	0.32
LOQ [µg/mL]	1.30	1.85	0.61	0.97	0.72	0.94
Regression Equation y = mx + c						
Slop [m]	0.0554	0.0242	0.0061	0.0022	0.0182	8.2964
Intercept [c]	−0.0099	0.0078	−0.0008	0.0003	0.0024	−0.7119
Correlation Coefficient [r^2^]	0.9999	0.9998	0.9995	0.9999	0.9999	0.9999

**Table 2:** Precision and accuracy data.

		Inter-day			Intra-day		
	Amount of Drug [µg/mL]	Amount found Mean [n = 3] ± SD	%RSD	%RE	Amount found Mean [n = 9] ± SD	%RSD	%RE
HPLC Method							
MOX	5	4.97 ± 0.05	1.01	−0.60	4.93 ± 0.07	1.42	−1.42
	100	101.2 ± 1.04	1.03	1.18	101.11 ± 1.5	1.57	1.10
	200	197.56 ± 3.25	1.65	−1.23	196.45 ± 2.33	1.19	−1.81
FLX	2	1.98 ± 0.01	0.51	−1.01	1.97 ± 0.02	1.02	−1.52
	100	99.87 ± 0.7	0.97	−0.13	100.15 ± 1.93	1.93	0.15
	200	196.89 ± 2.92	1.48	−1.58	197.07 ± 1.9	1.49	−1.49
First Derivative UV Spectroscopy							
MOX	2	1.97 ± 0.02	0.51	−1.52	1.98 ± 0.03	1.52	−1.01
	10	9.95 ± 0.14	1.41	−1.50	10.01 ± 0.16	1.60	0.10
	20	19.72 ± 0.37	1.88	−1.42	19.83 ± 0.38	1.92	−0.86
FLX	1	0.99 ± 0.01	1.01	−1.01	0.98 ± 0.01	1.02	−2.04
	8	7.96 ± 0.12	1.51	−0.50	7.91 ± 0.11	1.39	−1.14
	16	15.87 ± 0.27	1.70	−0.81	16.03 ± 0.22	1.37	0.19
Ratio First Derivative UV Spectroscopy							
MOX	1	0.98 ± 0.01	1.02	−2.04	0.99 ± 0.01	1.01	−1.01
	8	7.9 ± 0.09	1.14	−1.26	7.91 ± 0.15	1.90	−1.14
	16	15.75 ± 0.31	1.97	−1.58	15.76 ± 0.28	1.78	−1.52
FLX	1	0.99 ± 0.01	1.01	−1.01	1.01 ± 0.01	0.99	0.99
	8	7.89 ± 0.11	1.39	−1.39	8.03 ± 0.10	1.25	0.37
	16	15.77 ± 0.21	1.33	−1.45	15.82 ± 0.2	1.83	−1.14

**Table 3:** Determination of MOX and FLX from formulations and recovery studies by standard addition method.

Parameters	RP HPLC Method		First Derivative UV Method		Ratio First Derivative UV Method	
	Amount in [µg/mL]	% Recovery	Amount in [µg/mL]	% Recovery	Amount in [µg/mL]	% Recovery
Formulation [MOX]	80	98.67	6	99.18	8	101.29
Formulation [FLX]	40	99.03	3	100.89	4	98.47
Recovery of added MOX	40	100.1	3	101.67	4	98.75
	80	99.04	6	98.83	6	100.67
	120	98.71	9	98.67	8	98.88
	Across Mean	99.28		99.72		99.43
	%RSD	0.73		1.69		1.07
Recovery of added FLX	20	98.75	1.5	98.00	2	99.25
	40	99.08	3	101.33	3	98.67
	60	98.37	4.5	99.11	4	98.63
	Across Mean	98.73		99.48		98.85
	%RSD	0.35		1.70		0.34

## Supplementary Materials

The following are available online at https://www.mdpi.com/1660-4601/16/7/1196/s1, File 1: Effect of pH and Buffer concentration; File 2: Standard Calibration Curves.
