# Supplementary material for: Simultaneous Determination of Moxifloxacin and Flavoxate by RP-HPLC and Ecofriendly Derivative Spectrophotometry Methods in Formulations
Source: Int J Environ Res Public Health. 2019 Apr 3;16(7):1196. doi: 10.3390/ijerph16071196 (PMC6480697; doi:10.3390/ijerph16071196)
Supplement: Supplementary file 1 [file ijerph-16-01196-s001.zip › suppl/file 2--Standard Calibration Curves.docx]

**Simultaneous determination of moxifloxacin and flavoxate by RP HPLC and spectrophotometry methods in formulations**

**
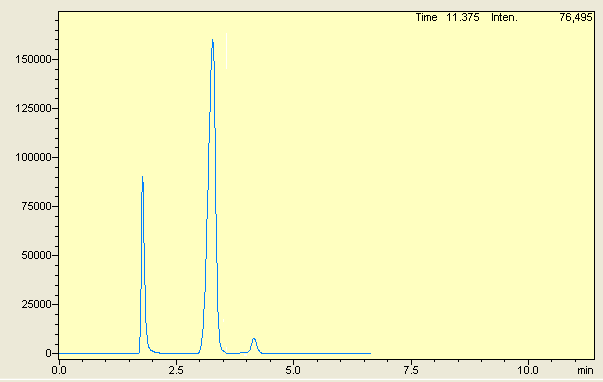
Chromatograms of standard solutions**

**Figure S1: Chromatogram of MOX (5 µg/ml), VST (50 µg/ml) and FLX (2 µg/ml)**

**
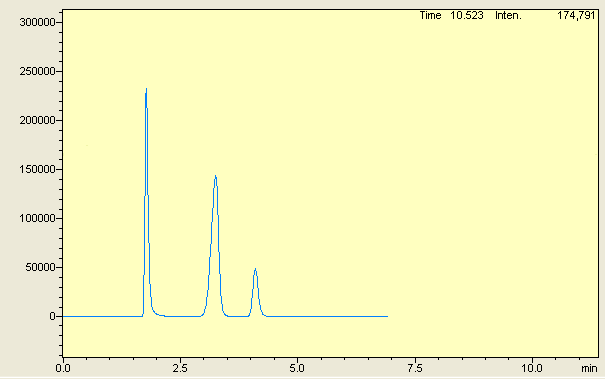
**

**Figure S2: Chromatogram of MOX (10 µg/ml), VST (50 µg/ml) and FLX (10 µg/ml)**

**
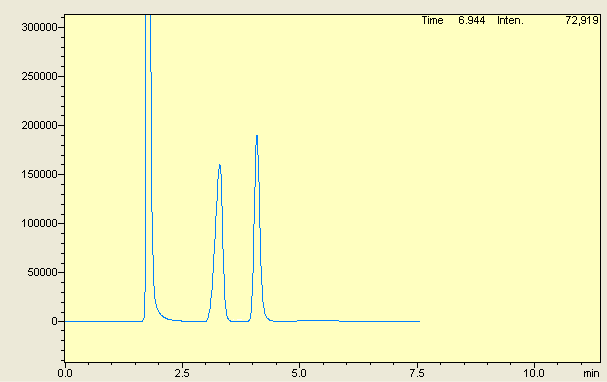
**

**Figure 3: Chromatogram of MOX (50 µg/ml), VST (50 µg/ml) and FLX (50 µg/ml)**

**
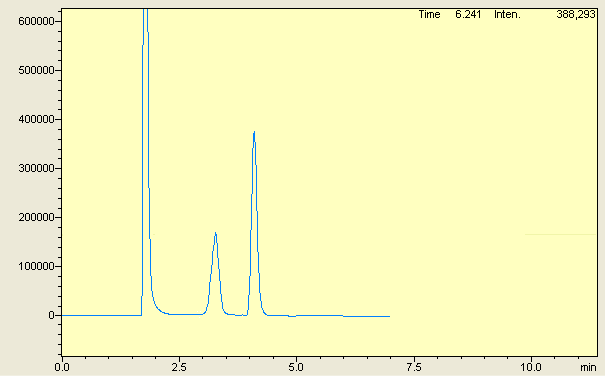
**

**Figure 4: Chromatogram of MOX (100 µg/ml), VST (50 µg/ml) and FLX (100 µg/ml)**

**
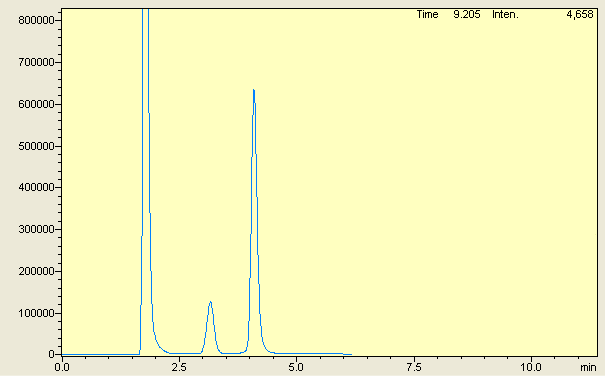
**

**Figure 5: Chromatogram of MOX (150 µg/ml), VST (50 µg/ml) and FLX (150 µg/ml)**

**
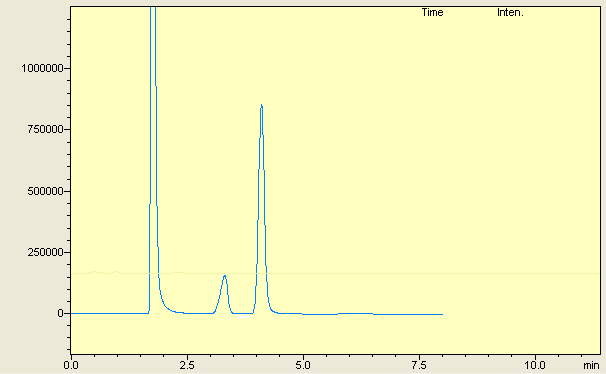
**

**Figure 6: Chromatogram of MOX (200 µg/ml), VST (50 µg/ml) and FLX (200 µg/ml)**

**Standard Calibration curves**

**
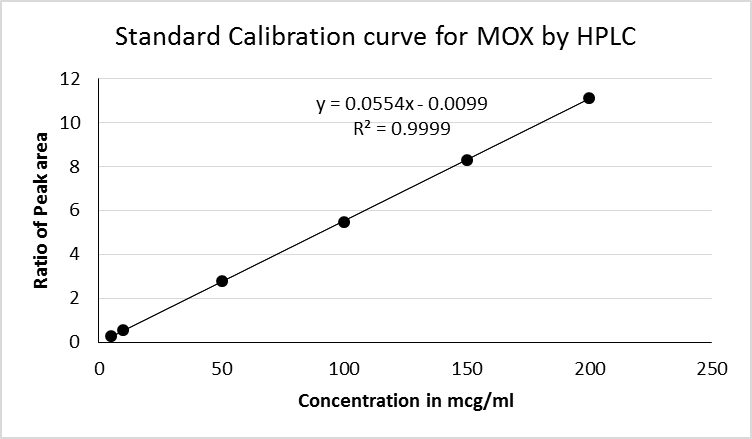
**

**Figure 7: Standard calibration curve for MOX by HPLC (5 to 200 mcg/ml)**

**
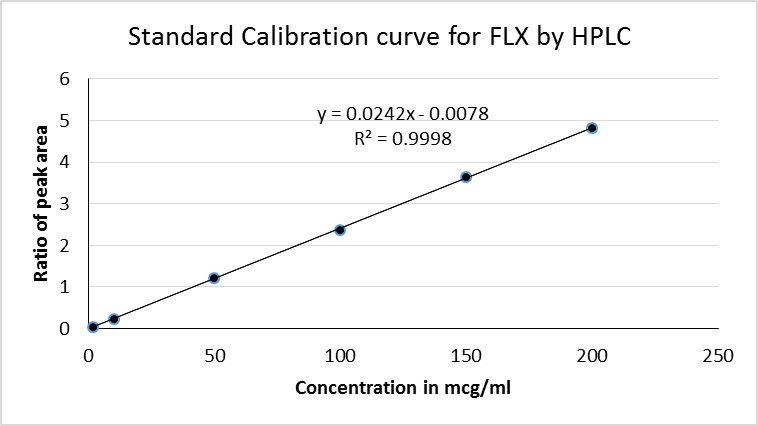
**

**Figure 8: Standard calibration curve for FLX by HPLC (2 to 200 mcg/ml)**

**
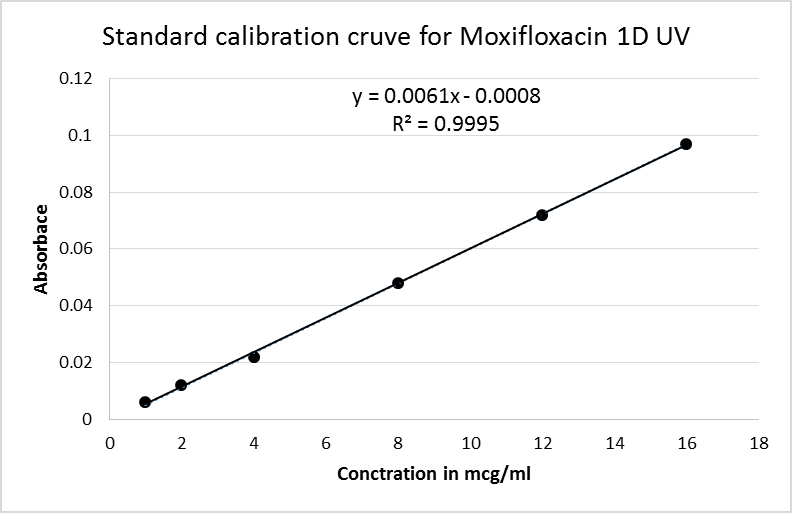
**

**Figure 9: Standard calibration curve for MOX by First derivation UV spctroscopy (1 to 16 mcg/ml)**

**
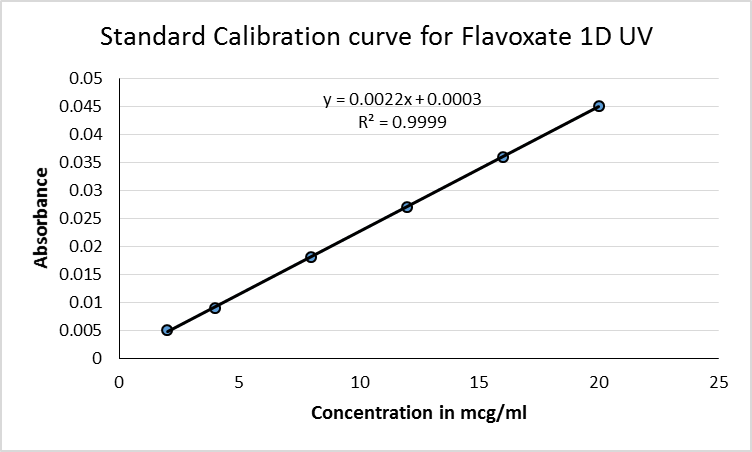
**

**Figure 10: Standard calibration curve for FLX by First derivation UV spctroscopy (2 to 20 mcg/ml)**

**
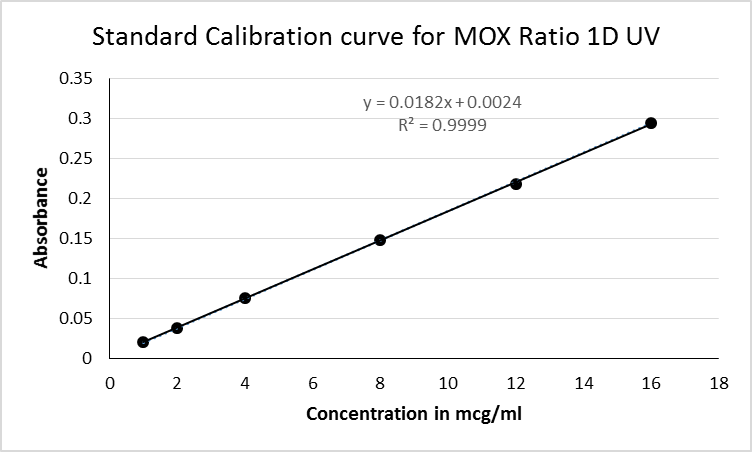
**

**Figure 11: Standard calibration curve for MOX ratio First derivation UV spctroscopy (1 to 16 mcg/ml)**

**
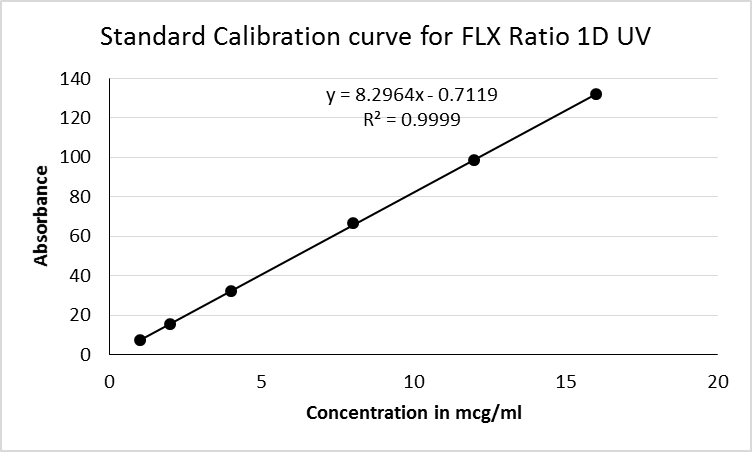
**

**Figure 12: Standard calibration curve for FLX by Ratio First derivation UV spctroscopy (1 to 66 mcg/ml)**
