# Supplementary material for: Simultaneous Determination of Moxifloxacin and Flavoxate by RP-HPLC and Ecofriendly Derivative Spectrophotometry Methods in Formulations
Source: Int J Environ Res Public Health. 2019 Apr 3;16(7):1196. doi: 10.3390/ijerph16071196 (PMC6480697; doi:10.3390/ijerph16071196)
Supplement: Supplementary file 1 [file ijerph-16-01196-s001.zip › suppl/file1--Effect of pH and Buffer concentration.docx]

**Simultaneous determination of moxifloxacin and flavoxate by RP HPLC and spectrophotometry methods in formulations**


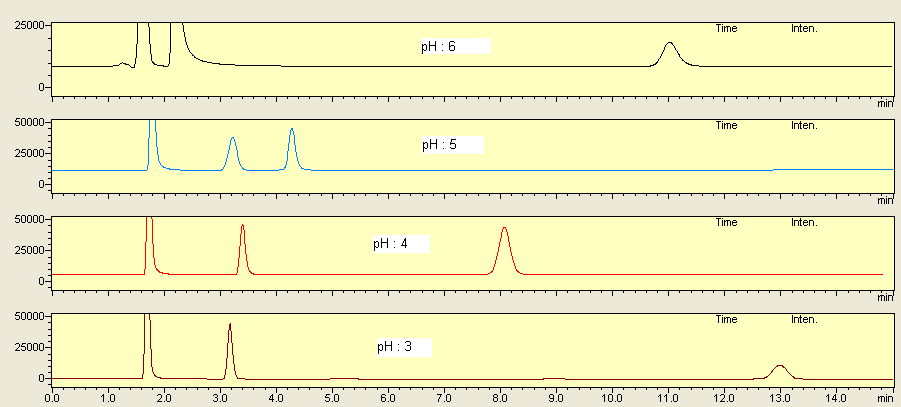


**Figure 1: Effect of pH on separation of MOX, FLX and VST**


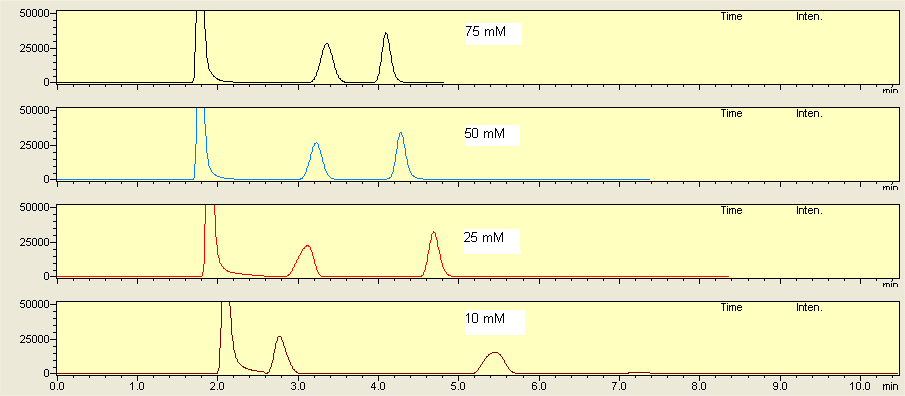


**Figure 2: Effect of phosphate concentration on separation of MOX, FLX and VST**
